# Identification of *Burkholderia cepacia* strains that express a *Burkholderia pseudomallei*-like capsular polysaccharide

**DOI:** 10.1128/spectrum.03321-23

**Published:** 2024-02-01

**Authors:** Mary N. Burtnick, David A. B. Dance, Manivanh Vongsouvath, Paul N. Newton, Sabine Dittrich, Amphone Sendouangphachanh, Kate Woods, Viengmon Davong, Dervla T. D. Kenna, Natnaree Saiprom, Sineenart Sengyee, Viriya Hantrakun, Vanaporn Wuthiekanun, Direk Limmathurotsakul, Narisara Chantratita, Paul J. Brett

**Affiliations:** 1 Department of Microbiology and Immunology, University of Nevada, Reno School of Medicine, Reno, Nevada, USA; 2 Department of Microbiology and Immunology, Faculty of Tropical Medicine, Mahidol University, Bangkok, Thailand; 3 Lao-Oxford-Mahosot Hospital-Wellcome Trust Research Unit, Microbiology Laboratory, Mahosot Hospital, Vientiane, Laos; 4 Nuffield Department of Medicine, Centre for Tropical Medicine and Global Health, University of Oxford, Oxford, United Kingdom; 5 Faculty of Infectious and Tropical Diseases, London School of Hygiene & Tropical Medicine, London, United Kingdom; 6 Deggendorf Institut of Technology, European Campus Rottal Inn, Pfarrkirchen, Germany; 7 Antimicrobial Resistance and Healthcare Associated Infections (AMRHAI) Reference Unit, Public Health Microbiology Division, Specialised Microbiology & Laboratories Directorate, UK Health Security Agency, London, United Kingdom; 8 Mahidol-Oxford Tropical Medicine Research Unit, Mahidol University, Bangkok, Thailand; 9 Department of Tropical Hygiene, Faculty of Tropical Medicine, Mahidol University, Bangkok, Thailand; University of Manitoba, Winnipeg, Manitoba, Canada

**Keywords:** *Burkholderia cepacia*, *Burkholderia pseudomallei*, *Burkholderia thailandensis*, capsular polysaccharide, melioidosis, monoclonal antibody

## Abstract

*Burkholderia pseudomallei* and *Burkholderia cepacia* are Gram-negative, soil-dwelling bacteria that are found in a wide variety of environmental niches. While *B. pseudomallei* is the causative agent of melioidosis in humans and animals, members of the *B. cepacia* complex typically only cause disease in immunocompromised hosts. In this study, we report the identification of *B. cepacia* strains isolated from either patients or soil in Laos and Thailand that express a *B. pseudomallei*-like 6-deoxyheptan capsular polysaccharide (CPS). These *B. cepacia* strains were initially identified based on their positive reactivity in a latex agglutination assay that uses the CPS-specific monoclonal antibody (mAb) 4B11. Mass spectrometry and *recA* sequencing confirmed the identity of these isolates as *B. cepacia* (formerly genomovar I). Total carbohydrates extracted from *B. cepacia* cell pellets reacted with *B. pseudomallei* CPS-specific mAbs MCA147, 3C5, and 4C4, but did not react with the *B. pseudomallei* lipopolysaccharide-specific mAb Pp-PS-W. Whole genome sequencing of the *B. cepacia* isolates revealed the presence of genes demonstrating significant homology to those comprising the *B. pseudomallei* CPS biosynthetic gene cluster. Collectively, our results provide compelling evidence that *B. cepacia* strains expressing the same CPS as *B. pseudomallei* co-exist in the environment alongside *B. pseudomallei*. Since CPS is a target that is often used for presumptive identification of *B. pseudomallei*, it is possible that the occurrence of these unique *B. cepacia* strains may complicate the diagnosis of melioidosis.

**IMPORTANCE:**

*Burkholderia pseudomallei*, the etiologic agent of melioidosis, is an important cause of morbidity and mortality in tropical and subtropical regions worldwide. The 6-deoxyheptan capsular polysaccharide (CPS) expressed by this bacterial pathogen is a promising target antigen that is useful for rapidly diagnosing melioidosis. Using assays incorporating CPS-specific monoclonal antibodies, we identified both clinical and environmental isolates of *Burkholderia cepacia* that express the same CPS antigen as *B. pseudomallei*. Because of this, it is important that staff working in melioidosis-endemic areas are aware that these strains co-exist in the same niches as *B. pseudomallei* and do not solely rely on CPS-based assays such as latex-agglutination, AMD Plus Rapid Tests, or immunofluorescence tests for the definitive identification of *B. pseudomallei* isolates.

## INTRODUCTION

Bacteria in the genus *Burkholderia* are widely distributed in the environment and commonly found in soils and surface waters worldwide (1). While many *Burkholderia* species play beneficial roles as free-living or host-associated microbes, a few cause disease in humans and animals. Of particular clinical importance are *Burkholderia pseudomallei*, the causative agent of melioidosis, and members of the *Burkholderia cepacia* complex that can colonize and infect immunocompromised hosts, especially cystic fibrosis and chronic granulomatous disease patients (1). Melioidosis is prevalent in tropical countries with the highest number of cases reported in Southeast Asia and northern Australia. *B. pseudomallei* infections are typically acquired through contact with contaminated soils or water and predominantly occur in individuals with underlying risk factors who reside in endemic areas (2). The clinical presentations of melioidosis are diverse and range from localized skin abscesses to acute pneumonia and sepsis, the latter of which can be rapidly fatal. Like most *Burkholderia* species, *B. pseudomallei* is naturally resistant to many commonly used antibiotics and no licensed melioidosis vaccines currently exist (3). Because of these issues, the rapid and accurate diagnosis of melioidosis is critical.

The current gold standard method for diagnosing melioidosis is the culture and identification of *B. pseudomallei* from clinical samples. This is time- and labor-intensive and lacks sensitivity (4). In order to reduce the time to obtain a presumptive identification, various approaches have been used, including latex agglutination with a monoclonal antibody (mAb) 4B11 against the 6-deoxyheptan capsular polysaccharide (CPS) (5). This approach has been used in clinical and research laboratories in Thailand and Laos for many years, and although cross-reactions with other *Burkholderia* species from environmental samples have been reported, this has not previously been reported among clinical isolates (6). Such presumptive misidentifications are inevitably misleading and might result in patients being treated inappropriately for melioidosis unless the error is recognized.

The 6-deoxyheptan CPS expressed by *B. pseudomallei* is a key virulence determinant encoded by a 34.5-kb gene cluster located on chromosome I (7, 8). Experimental evidence has shown that CPS reduces phagocytosis of *B. pseudomallei* by host cells, preventing complement factor C3b deposition on the bacterial surface (9). Several studies indicate that CPS is a promising vaccine candidate and that antibodies against CPS provide protection in animal models of melioidosis (10–13). CPS is also considered an attractive antigen for the development of rapid point-of-care diagnostics since it is highly conserved among *B. pseudomallei* isolates and is known to be shed and circulate throughout host tissues during active infections (14, 15). In addition to the latex agglutination assay mentioned above, lateral flow immunoassays (LFIs) such as the Active Melioidosis Detect (AMD, InBios International, Inc.) assay that employs the CPS-specific mAb 4C4 have been developed and tested using a variety of *Burkholderia* isolates and clinical samples and have been shown to be highly specific for *B. pseudomallei* (16–18).

*Burkholderia thailandensis* is a closely related, non-pathogenic, near-neighbor species that co-exists in the same environmental niches as *B. pseudomallei* (19, 20). While lack of 6-deoxyheptan CPS production was once considered to be a key differentiating feature between *B. thailandensis* and *B. pseudomallei*, several recent studies have identified *B. thailandensis* variants that express a *B. pseudomallei*-like CPS (21–26). *B. thailandensis* E555, a soil isolate from Cambodia, is the most well-characterized CPS-expressing variant strain to date. E555 has been shown to harbor a highly similar CPS biosynthetic gene cluster to that of *B. pseudomallei* and expresses a structurally identical CPS; however, this isolate is avirulent in mice (21, 23). Although *B. thailandensis* infections have been reported in humans, these appear to be exceedingly rare (24, 27–29).

In this study, we report the identification and characterization of three *B. cepacia* isolates that produce a *B. pseudomallei*-like CPS along with two previously undescribed CPS-expressing *B. thailandensis* strains. Importantly, two of the CPS-expressing *B. cepacia* strains are clinical isolates that were identified in areas where *B. pseudomallei* is endemic.

## MATERIALS AND METHODS

### Strains

The *B. cepacia* and *B. thailandensis* strains used in this study are described in Table 1. All strains were obtained from the Lao-Oxford-Mahosot Hospital-Wellcome Trust Research Unit (LOMWRU) Microbiology Laboratory strain collection in Vientiane, Laos, or the Mahidol-Oxford Tropical Medicine Research Unit (MORU) strain collection at Mahidol University, Bangkok, Thailand. Work with *B. pseudomallei* strain K96243 (7) was conducted in CDC-registered biosafety level 3 (BSL-3) facilities in compliance with the rules and regulations of the U.S. Federal Select Agent Program.

**TABLE 1:** *B. cepacia* and *B. thailandensis* strains used in this study

Species	Strain	Sample type	Country of isolation	Latex agglutination result	Purified total carbohydrate yield (from 1 L of culture)
*B. cepacia*	LNT40	Environment	Laos	+	27.1 mg
*B. cepacia*	39628	Clinical	Laos	+	10.2 mg
*B. cepacia*	10223	Clinical	Thailand	+	20.4 mg
*B. cepacia*	2.1B	Soil	Thailand	−	25 mg
*B. cepacia*	U668	Clinical	Thailand	−	26.8 mg
*B. thailandensis*	E555^*a*^	Soil	Cambodia	+	8.8 mg
*B. thailandensis*	SBXCC001	Soil	Thailand	+	9.5 mg
*B. thailandensis*	SBXPR001	Soil	Thailand	+	8.8 mg
*B. pseudomallei*	K96243	Clinical	Thailand	ND^*b*^	ND^*b*^

^*a*^ Previously described by Sim et al. (23).

^*b*^ ND, not determined in this study.

### Clinical isolates

*B. cepacia* strain 39628 was collected in Laos as a routine diagnostic specimen in 2014 with oral consent. *B. cepacia* strain 10223 was obtained from a sputum sample in 2010 and was stored anonymously as per routine research lab practice at the time. *B. cepacia* strain U668 was collected from a patient in NE Thailand in 1990 and was stored anonymously as part of routine lab practice at that time. The researchers did not have access to information that could identify individual participants after strain collection.

### Latex agglutination assays

The latex agglutination assay is based on a specific reaction between mAb 4B11 and CPS of *B. pseudomallei*. The latex agglutination reagent was prepared by Mahidol University and used for testing as previously described (30). Briefly, the assay was performed by mixing single bacterial colonies with 10 µL of latex reagent on a glass slide. Agglutination was observed by eye within 1–2 min after mixing.

### Biochemical tests and antibiograms

Isolates in Laos were presumptively identified as species using the API 20NE (bioMérieux) kit according to the manufacturer’s instructions, and antimicrobial susceptibility tests were conducted by disk diffusion according to the methods of the Clinical Laboratory Standards Institute that were current at the time, interpreted according to local guidelines (31).

### DNA sequencing and polymerase chain reaction

#### *16s RNA* sequencing

For the 16s RNA assay, primers 16SU17F and 16s 1541R (PH) were used and adapted from Edwards et al. (32). Purified (Qiagen, Germany) amplicons of ~1.5 kbp were sequenced (Macrogen, Korea) and analyzed using NCBI databases to confirm the species.

#### *recA* sequencing

For the *recA* polymerase chain reaction (PCR) assay, primers BCR1 and BCR2 were used, as described by Mahenthiralingham et al. (33). For *recA* sequencing, these primers were combined with two others: Bcc seqF1 and Bcc seqR2, as described by Turton et al. (34). BioNumerics was used to analyze the sequences, which were clustered with type and reference strains using the neighbor-joining method.

### Matrix-assisted laser desorption/ionization biotyping

Bacterial isolates were prepared and analyzed by the matrix-assisted laser desorption/ionization (MALDI) Biotyper system as previously described (35). Briefly, all isolates were cultured on Columbia agar at 37°C under aerobic conditions for 24 h, extracted with formic acid, and 1 µL of supernatant was spotted onto an MSP-384 polished steel target plate (Bruker Daltonics, Germany) and dried. Following this, 1 µL of a saturated solution of MALDI matrix, α-cyano-4-hydroxycinnamic acid (Bruker Daltonics, Germany) was applied to each sample and dried. Measurements were performed with the Bruker MALDI Biotyper system using FlexControl software (version 3.4.135; Bruker Daltonics, Germany). Spectra ranging from 2,000 to 13,000 *m*/*z* were analyzed using the MALDI-Biotyper software (version 3.1; Bruker Daltonics, Germany) and a reference database supplemented with a *Burkholderia* library (35). An identification score of ≥2.3 indicated reliable species identification, a score of 2.0–2.29 indicated probable species level identification, a score of 1.7–1.9 indicated probable genus level identification, and a score of <1.7 indicated no reliable identification (35).

### Carbohydrate purification

*B. cepacia* and *B. thailandensis* strains were grown overnight in 1 L of Luria Bertani-Lennox (LBL) broth at 37°C with aeration. Bacterial cultures were pelleted by centrifugation (10 min at 8,000 × *g*) and the resulting cell pellets were extracted using a modified hot aqueous-phenol procedure essentially as previously described (36, 37). Following extraction, the phenol and aqueous phases were combined and dialyzed against distilled water to remove the phenol. The dialysates were clarified by centrifugation (10 min at 10,000 × *g*) and the supernatants were concentrated by lyophilization. The samples were then treated with RNase A, DNase I, and proteinase K (50 µg/mL), and the resulting carbohydrates were isolated as precipitated gels following successive rounds of ultracentrifugation. The gel-like pellets were resuspended in ultrapure water, lyophilized, and weighed to determine the yield of total carbohydrate.

### CPS-specific lateral flow immunoassays

Active Melioidosis Detect (AMD) Plus Rapid Tests (InBios International, Inc.) were kindly provided for clinical work at LOMWRU and used following the manufacturer’s instructions. The AMD Plus Rapid Tests were purchased for research use at the University of Nevada, Reno, and used per the manufacturer’s instructions. Purified total carbohydrate samples (1 µg) from five *B. cepacia* strains and one *B. thailandensis* strain were individually loaded onto rapid tests followed by chase buffer. Results were determined after incubation at room temperature for 15 min.

### SDS-PAGE, Western immunoblotting, and silver staining

Purified carbohydrate samples resuspended in water were mixed 1:1 with 2× SDS-PAGE sample buffer and heated to 100°C for 5 min prior to electrophoresis on 12% Tris-glycine gels (Invitrogen). For immunoblot analysis, the antigens were electrophoretically transferred to nitrocellulose membranes. The membranes were blocked with 3% skim milk in high-salt Tris-buffered saline (HS-TBS; 20 mM Tris, 500 mM NaCl, pH 7.5) for 60 min at room temperature and then incubated for 60 min at room temperature with a 1/1,000 or 1/2,000 dilution of a CPS-specific mAb (3C5, 4C4, or MCA135) or with a 1/400 dilution of a type A lipopolysaccharide (LPS)-specific mAb (Pp-PS-W). To facilitate detection, the membranes were incubated for 60 min at room temperature with a 1/5,000 dilution of anti-mouse IgG or IgM horseradish peroxidase conjugates (Southern Biotech). The blots were visualized using Pierce ECL Western Blotting Substrate (Thermo Scientific) and a ChemiDoc XRS imaging system (BioRad). CPS purified from *B. pseudomallei* RR2683 was used as a positive control (11).

For analysis of LPS, the purified carbohydrate samples were electrophoresed on 12% Tris-glycine gels as described above. Silver staining was conducted essentially as previously described (38).

### Genomic DNA isolation, genome sequencing, and analysis

*B. cepacia* (strains LNT40, 39628, and 10223) and *B. thailandensis* (strains SBXCC001 and SBXPR001) were grown overnight in LBL broth at 37°C with aeration. DNA was extracted from the strains using a Wizard Genomic DNA Purification Kit (Promega) as per the manufacturer’s instructions. DNA preparations were further purified by ethanol precipitation using a standard protocol. Sequencing of the genomic DNA samples was conducted at the Institute for Genome Sciences (IGS) Genomics Resource Center (Baltimore, MD, USA). PacBio single-molecule real-time (SMRT) sequencing was conducted on a PacBio RS II instrument to ~16× coverage (strains 39268 and LNT40) or ~24× coverage (strains 10223, SBXCC001, and SBXPR001) using 20 kb SMRTbell libraries and P6C4 chemistry. PacBio genomic data were assembled using the Hierarchical Genome Assembly Process algorithm version 3 (10) implemented in PacBio SMRT Portal version 2.3.0 for (strains 39268 and LNT40) (39) or Celera Assembler version 8.2 (strains 10223, SBXCC001, and SBXPR001) (40). Assemblies were reorganized relative to the *B. pseudomallei* K96243 genome (41).

The IGS Annotation Engine was used for structural and functional annotation of the sequences (https://ae.igs.umaryland.edu [42]). Manatee was used to view annotations (http://manatee.sourceforge.net/). Submission of the genomes to GenBank and comparative analysis of the annotated genomes were conducted by the IGS Informatics Resource Center (University of Maryland). Sequence Read Archive (SRA) and GenBank accession numbers for each genome are listed in Table 2.

**TABLE 2:** Genome characteristics of *B. cepacia* and *B. thailandensis* strains sequenced in this study

Species	Strain name	Source	Genome size (bp)	No. of chromosomes (size)	No. of plasmids (size)	% G+C	Location of CPS gene cluster (locus tags)	GenBank accession no.	SRA accession no.
*B. cepacia*	LNT40	Environmental	8,585,420	3: contig.0_1 (3,670,428 bp), contig.1_1 (1,309,270 bp), contig.2_1 (3,605,722 bp)	0	66.7	contig.0_1: 232284–266734 (C7S13_0262–C7S13_0285)	SAMN08724753	SRS20034251
*B. cepacia*	39628	Clinical	8,403,907	3: contig.0_1 (3,683,734 bp), contig.1_1 (1,304,848 bp), contig.2_1 (3,415,325 bp)	0	66.8	contig.0_1: 268641–303096 (C7S14_3743–C7S14_3766)	SAMN08724754	SRS20041683
*B. cepacia*	10223	Clinical	8,703,468	3: contig.0_1 (3,848,587 bp), contig.1_1 (3,329,779 bp), contig.3_1 (1,312,533 bp)	1 contig.2_1 (212,569 bp)	66.4	contig.0_1: 990599–1025048 (C7S15_1049–C7S15_1072)	SAMN08724755	SRS20034250
*B. thailandensis*	SBXCC001	Environmental	6,842,881	2: contig.0_1 (3,018,887 bp), contig.1_1 (3,823,994 bp)	0	67.7	contig.1_1: 1809273–1843756 (C7S16_1973–C7S16_2004)	SAMN08724756	SRS20034253
*B. thailandensis*	SBXPR001	Environmental	7,015,772	2: contig.0_1(3,095,076 bp), contig.1_1 (3,920,696 bp)	0	67.5	contig.1_1: 2170112–2204606 (C7S17_2372–C7S17_2396)	SAMN08724757	SRS20034727

For comparison of the CPS operons, the three *B. cepacia* and two *B. thailandensis* genomes including the reference genome *B. pseudomallei* K96243 (NC_006350) were run through a comparative analysis pipeline to generate protein ortholog clusters using Jaccard-filtered bi-directional best blast matches. Sybil (http://sybil.sourceforge.net/documentation.html), a web-based graphical user interface, was used to search and view ortholog clusters. The genomic comparative view pictures of the clusters in the CPS operon region were generated by selecting the genomes of interest.

### CPS-specific immunofluorescence assay

Immunofluorescence assays (IFAs) using the CPS-specific mAb 4B11 were conducted essentially as previously described (43). Briefly, bacteria were cultured in LB broth at 37°C overnight following which 1 mL of culture was centrifuged at 10,000 rpm for 5 min, washed three times with PBS, and fixed with 500 µL of 2% paraformaldehyde in PBS for 15 min. The fixed bacteria were washed again with PBS and stained with IFA reagents (containing mAb 4B11 and Alexa Fluor 488 conjugated-goat anti-mouse IgG at a dilution of 1:1,000 in PBS) for 20 min at room temperature. Bacteria were observed using a laser scanning confocal microscope (LSM 700; Carl Zeiss) using a 100× objective lens with oil immersion and Zen software (2010 edition, Zeiss, Germany).

## RESULTS

### Identification of *B. cepacia* strains testing positive in the melioidosis latex agglutination test

The first cross-reacting *B. cepacia* isolate (LNT40) was recognized during the re-examination of isolates from an environmental study undertaken in Laos in 2009 (44). Although this isolate agglutinated strongly with the anti-CPS monoclonal antibody-based latex reagent and gave a strong positive reaction with the AMD test (17), the colony morphology was atypical. Subsequent examination by API 20NE, antibiogram (specifically resistance to co-amoxiclav), 16S rDNA sequencing, and PCR to distinguish between *B. pseudomallei*, *B. thailandensis,* and *B. cepacia* (45) suggested that it was actually a member of the *B. cepacia* complex (data not shown).

In 2014, an oxidase-positive Gram-negative bacillus that agglutinated strongly with the latex reagent was isolated at Mahosot Hospital from the sputum of an outpatient. Further examination of the isolate (designated strain 39628) confirmed the agglutination reaction but API 20NE and antibiogram suggested that the isolate was *B. cepacia* (data not shown). Testing of 32 further clinical isolates of *B. cepacia* complex from the LOMWRU collection revealed that strains LNT40 and 39628 were the only two isolates that gave this cross-reaction. In addition, DNA from these 32 isolates was extracted and sent to the Antimicrobial Resistance and Healthcare Associated Infections Reference Laboratory (AMRHAI), London, UK, for the identification of specific genomovars by *recA* sequencing. Both LNT40 and 39628 were identified as *B. cepacia* (genomovar I) as were four of the other non-cross-reacting isolates. Of the other strains tested, 25 were identified as *B. cenocepacia* IIIA, one each was *B. seminalis* and Taxon K and one was unassignable (data not shown).

### MALDI Biotyper analysis of *B. cepacia* strains

**Fig 1:**
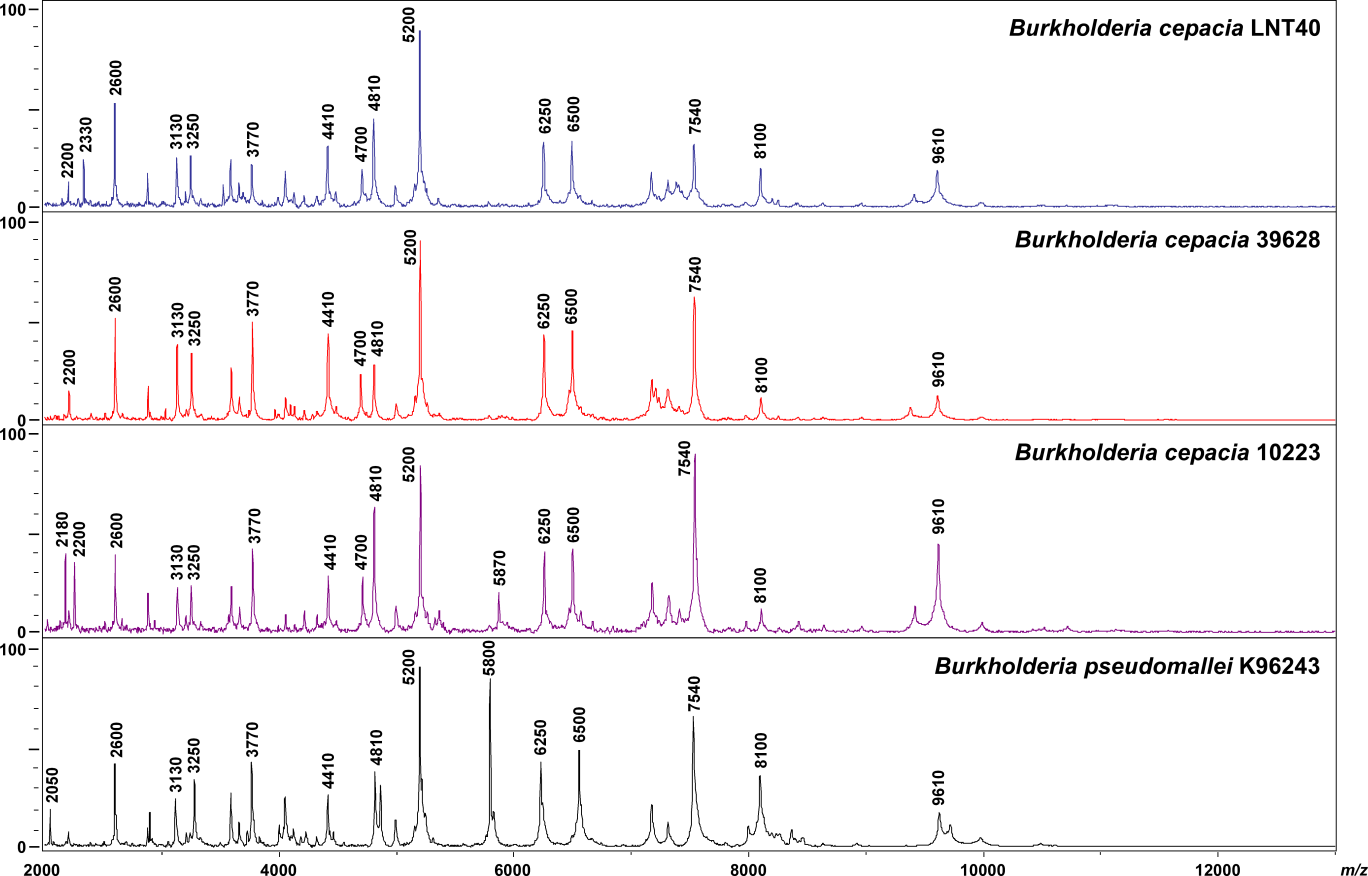
MALDI-TOF spectra of *B. cepacia* strains LNT40, 39628, 10223 and *B. pseudomallei* strain K96243. The characteristic spectra of the bacterial isolates were generated by MALDI-TOF MS. The relative intensities of ions are shown on the *y* axis, and the mass to charge ratio (*m*/*z*) is shown on the *x* axis.

To further characterize the two cross-reacting *B. cepacia* isolates (LNT40 and 39628) from the LOMWRU collection, MALDI biotyping experiments were conducted. For comparative purposes, a Thai clinical isolate of *B. cepacia* (strain 10223) that cross-reacted with the latex reagent obtained from the MORU collection and *B. pseudomallei* strain K96243 were also included in this analysis. The three *B. cepacia* isolates (strains LNT40, 39628, and 10223) and *B. pseudomallei* strain K96243 were subjected to MALDI-TOF-MS and analyzed using MALDI Biotyper system software with a supplemented *Burkholderia* reference database. Results showed that all of the latex-positive *B. cepacia* isolates were identified as belonging to the *B. cepacia* complex (score values ≥ 2.300) as opposed to the *B. pseudomallei* complex (Table 3). The three isolates of *B. cepacia* also demonstrated similar protein profile patterns. Peaks were observed at approximately *m*/*z* of 2,600, 2,880, 3,130, 3,250, 3,770, 4,410, 4,810, 5,200, 6,250, 6,500, 7,540, 8,100, and 9,610 for all *B. cepacia* strains and *B. pseudomallei* (Fig. 1). Importantly, peaks that were unique to the *B. cepacia* isolates were at approximately *m/z* of 2,200 and 4,700 and peaks at *m*/*z* of 2,050 and 5,800 were only observed in *B. pseudomallei*. In addition, a peak at *m*/*z* of 2,330 was only observed in *B. cepacia* strain LNT40 while peaks at *m*/*z* 2,180 and 5,870 were found only in *B. cepacia* strain 10223. Taken together, these data are consistent with the results of the latex agglutination assays, API 20NE tests, antibiograms, *recA* and 16S sequencing, and PCR tests and support the conclusion that strains LNT40 and 39628 are isolates of *B. cepacia* rather than *B. pseudomallei*.

**TABLE 3:** Identification of *B. cepacia* and *B. pseudomallei* by Bruker MALDI Biotyper system

Species	Strain	Identification results by Bruker MALDI Biotyper system^*a*^	Score value^*b*^
*B. cepacia*	LNT40	*B. cepacia*	2.420
*B. cepacia*	39628	*B. cepacia*	2.442
*B. cepacia*	10223	*B. cepacia*	2.516
*B. pseudomallei*	K96243	*B. pseudomallei*	2.575

^*a*^ Extended reference profile database for *Burkholderia* species.

^*b*^ Score value > 2.3 indicates species identification.

### Reactivity of *B. cepacia* strains with CPS-specific mAbs

**Fig 2:**
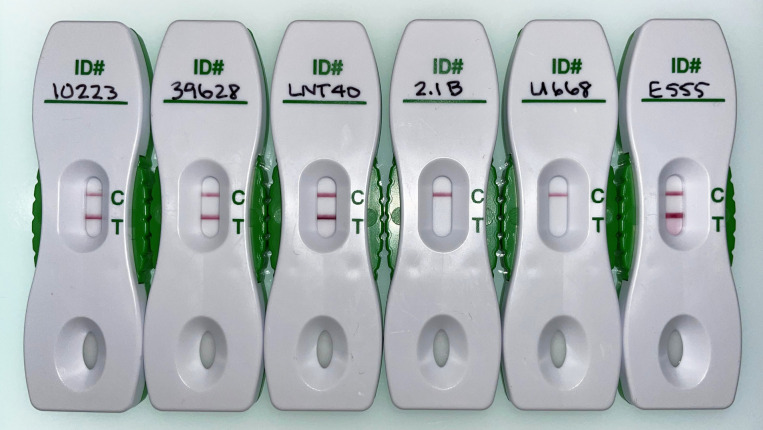
Analysis of purified carbohydrate samples using Active Melioidosis Detect (AMD) Plus Rapid Tests. Each AMD Plus test was loaded with 1 µg of purified total carbohydrate from *B. cepacia* strains LNT40, 39628, 10223, 2.1B, U668, and *B. thailandensis* strain E555. The test results were captured after 15 min of incubation at room temperature. The control line is indicated by “C” and the test line by “T.” Samples with a line at both positions are considered positive for CPS. *B. thailandensis* strain E555 was used as a positive control.

Total carbohydrate was extracted from *B. cepacia* strains LNT40, 39628, and 10223 along with one clinical and one soil isolate of *B. cepacia* (strains 2.1B and U668) that tested negative in the latex agglutination assay. In addition, three soil isolates of *B. thailandensis* (strains E555, SBXCC001, and SBXPR001) had also been found to agglutinate with the latex reagent and/or were known to express the 6-deoxyheptan CPS antigen (21–23). The yields of total carbohydrate obtained from these strains ranged from 8.8 to 27.1 mg/L and are shown in Table 1. To determine if the purified carbohydrate samples contained the CPS antigen of interest, they were tested with AMD Plus LFIs that use the CPS-specific mAb 4C4 (17). As expected, *B. cepacia* strains LNT40, 39628, and 10223, as well as *B. thailandensis* strains E555, SBXCC001, and SBXPR001 exhibited positive results on the AMD Plus tests while *B. cepacia* strains 2.1B and U668 were negative (Fig. 2).

**Fig 3:**
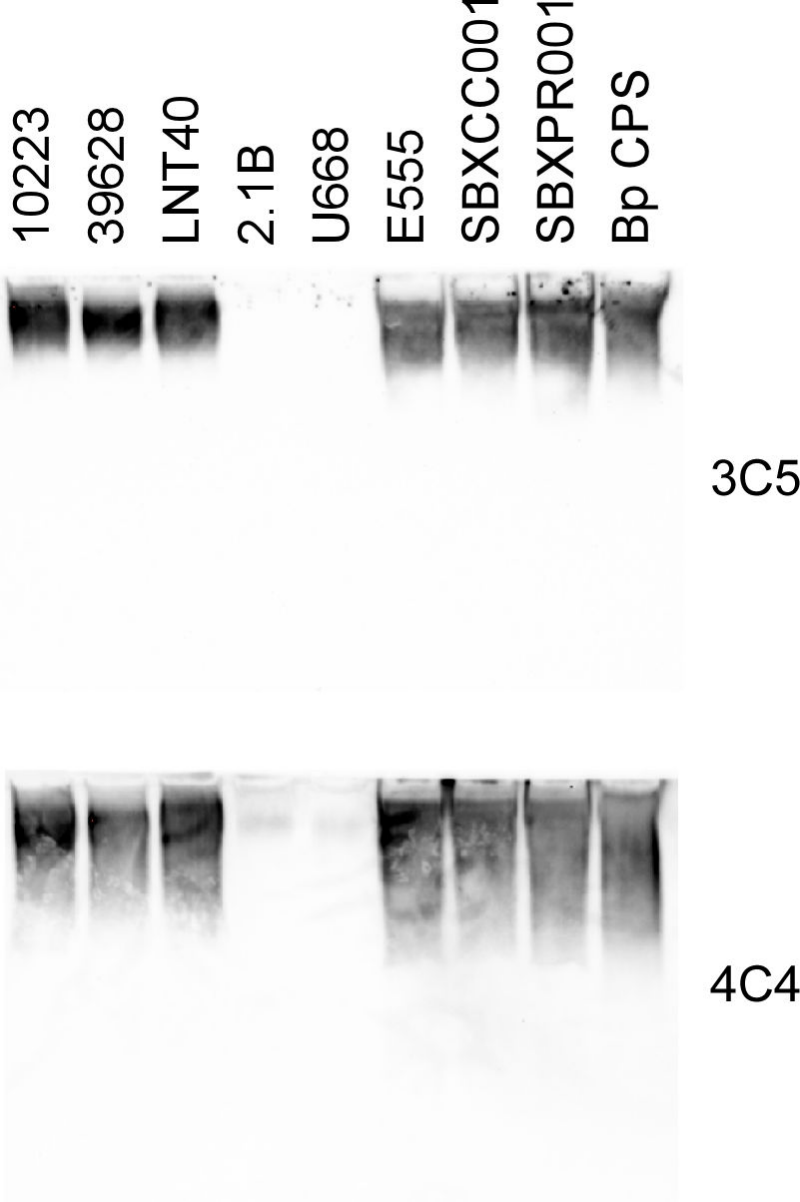
Western immunoblot analysis of purified carbohydrate samples. Purified carbohydrates from *B. cepacia* strains 10223, 39628, LNT40, 2.1B, and U668 (10 µg/lane) and *B. thailandensis* strains E555, SBXCC001, and SBXPR001 (2 µg/lane) were probed with CPS-specific mAbs 3C5 and 4C4. Purified *B. pseudomallei* RR2683 (Bp) CPS (1 µg/lane) was used as a positive control.

To further characterize the total carbohydrate samples extracted from the *B. cepacia* and *B. thailandensis* strains, three different CPS-specific mAbs (3C5, 4C4, and MCA147) were used in Western immunoblot analyses. As shown in Fig. 3, *B. cepacia* strains LNT40, 39628, and 10223 and *B. thailandensis* strains E555, SBXCC001, and SBXPR001 reacted strongly with mAbs 3C5 and 4C4, but *B. cepacia* strains 2.1B and U668 did not. Similar results were observed with mAb MCA147 (data not shown). These findings were consistent with the results of both the latex agglutination and AMD Plus immunoassays.

**Fig 4:**
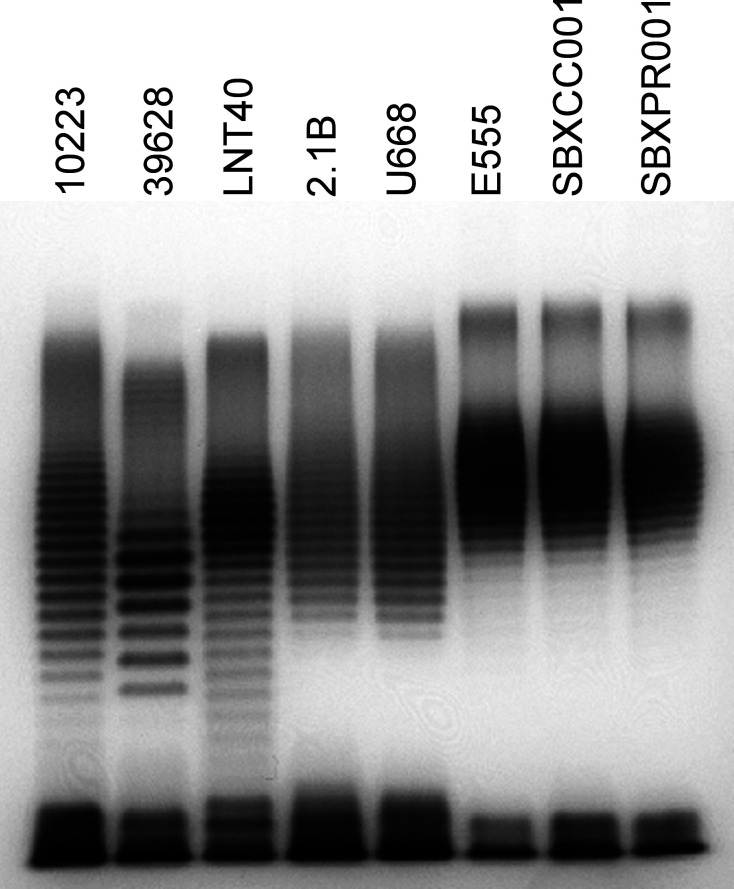
Silver stain analysis of purified carbohydrate samples. Purified carbohydrates from *B. cepacia* strains 10223, 39628, LNT40, 2.1B, and U668 and *B. thailandensis* strains E555, SBXCC001, and SBXPR001 (5 µg/lane) were separated on a 12% Tris-glycine gel and visualized by silver staining.

To examine the LPS expressed by the *B. cepacia* and *B. thailandensis* strains, SDS-PAGE and silver staining were conducted on all of the purified carbohydrate samples. As shown in Fig. 4, *B. cepacia* strains 2.1B and U668 displayed LPS banding patterns that were similar to one another as did the three *B. thailandensis* strains. In contrast, the three CPS-expressing *B. cepacia* strains appeared to have unique LPS banding patterns that were different from the other strains tested, with strains 10223 and LNT40 appearing similar to one another. To determine if the LPS moieties expressed by *B. cepacia* and *B. thailandensis* strains could be recognized by the *B. pseudomallei* type A LPS-specific mAb (Pp-PS-W), Western immunoblotting was conducted. Results demonstrated that only the *B. thailandensis* strains reacted with the mAb (data not shown).

### Genome sequencing of the latex agglutination positive *B. cepacia* strains

To determine whether the CPS-expressing *B. cepacia* isolates LNT40, 39628, and 10223 harbored the genes necessary for CPS expression in *B. pseudomallei*, whole genome sequencing was conducted. The genomes of two CPS-expressing *B. thailandensis* strains SBXCC001 and SBXPR001 were also sequenced for comparative purposes. The genome characteristics of each of the strains sequenced are shown in Table 2. All three *B. cepacia* strains harbored three chromosomes each, with genome sizes totaling ~8.4 to 8.7 Mb with G+C contents of ~66.4% to 66.8%. Interestingly, strain 10223 also harbored a plasmid of ~212 kb. The two *B. thailandensis* strains each harbored two chromosomes with overall genome sizes of ~6.8 and ~7 Mb with G+C contents of ~67.7% and ~67.5%, respectively.

**Fig 5:**
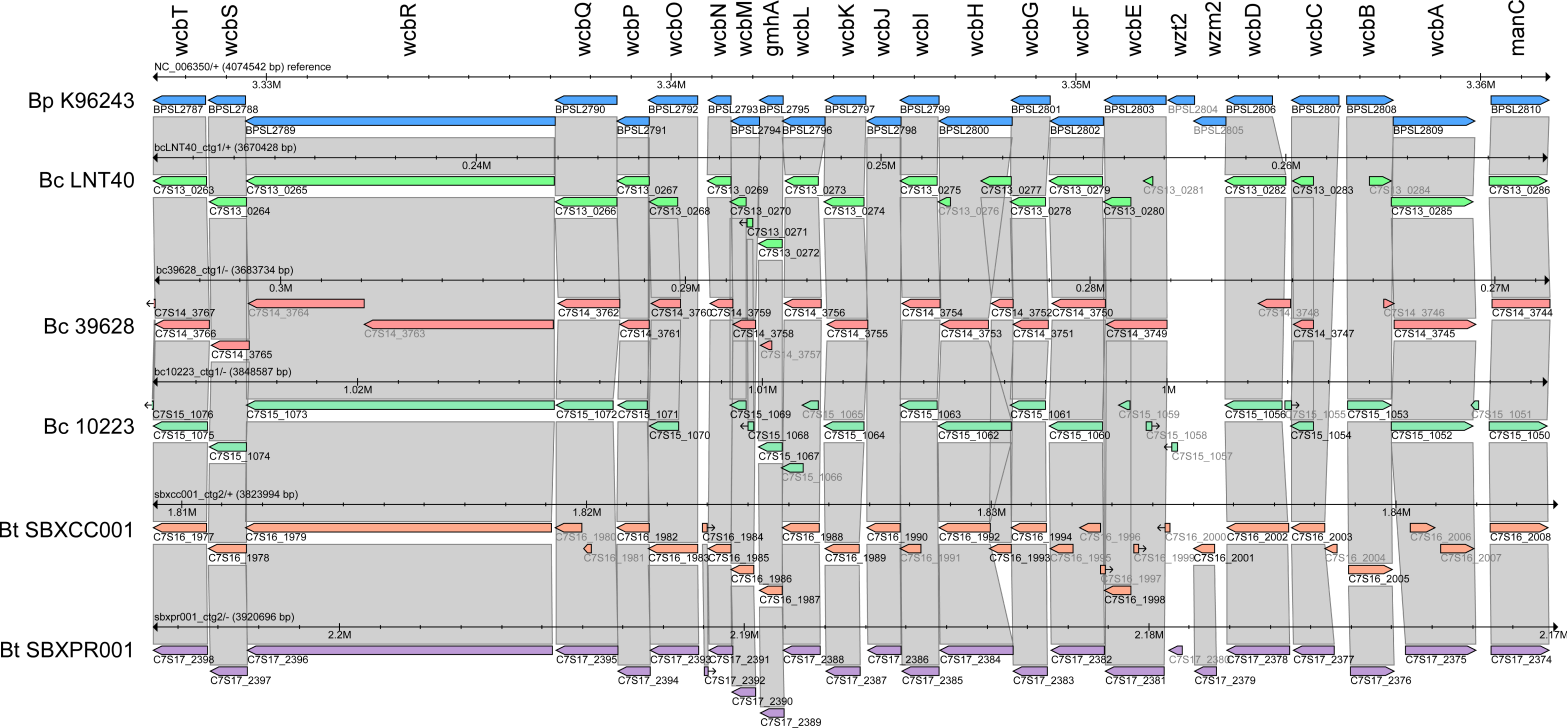
Alignment of CPS biosynthesis gene clusters. Figure showing a 34.5-kb region of *B. pseudomallei* (Bp) K96243 containing 24 genes (BPSL2787 to BPSL2810, *wcbT-manC*) responsible for CPS biosynthesis. Similar regions in *B. cepacia* (Bc) strains 10223, 39628, LNT40, and *B. thailandensis* (Bt) strains SBXCC001 and SBXPR001 are aligned below the reference genome. Locus tags are shown below each of the open reading frames identified.

To determine if homologs of the *B. pseudomallei* CPS biosynthesis genes were present in the CPS-expressing *B. cepacia* and *B. thailandensis* strains, the K96243 CPS gene cluster (locus tags BPSL2787-BPSL2810) was used as a reference. The 34.5 kb region of *B. pseudomallei* K96243 containing 24 genes responsible for CPS biosynthesis was compared to the genome sequences of *B. cepacia* strains LNT40, 39628, and 10223 and *B. thailandensis* strains SBXCC001 and SBXPR001. The resulting alignments are shown in Fig. 5, and the location of CPS gene clusters (locus tags) for each of the strains is listed in Table 2. While homologs for the majority of CPS biosynthesis genes were identified in the *B. cepacia* and B. *thailandensis* strains, some genes were notably absent. For example, *wzt2* and *wzm2* that encode for a putative ABC transporter involved in CPS export were both absent from the *B. cepacia* strains, and were truncated in the *B. thailandensis* strains.

**Fig 6:**
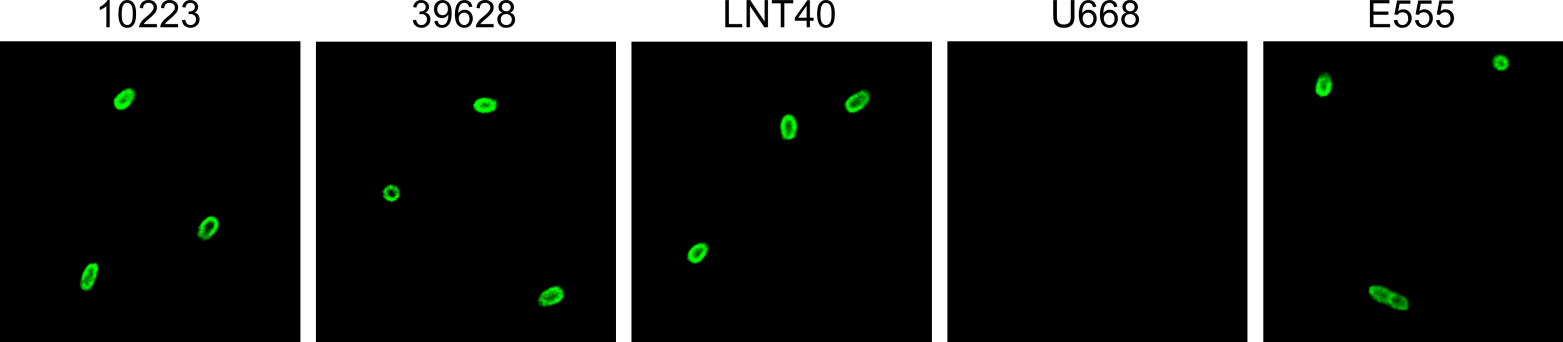
IFAs using CPS-specific mAb 4B11. Fluorescent microscopy of *B. cepacia* (strains 10223, 39628, LNT40, and U668) and *B. thailandensis* (strain E555) stained with mAb-IFA reagent.

To determine if the CPS antigen was expressed on the surface of the latex-positive *B. cepacia* strains, IFAs based on the CPS-specific mAb 4B11 were conducted. As shown in Fig. 6, *B. cepacia* strains 10223, 39628, LNT40, and *B. thailandensis* strain E555 demonstrated robust fluorescence, while *B. cepacia* U668 did not. These findings are consistent with the latex agglutination assay results, the LFI and Western immunoblotting results, and the presence or absence of CPS biosynthetic genes in these strains.

## DISCUSSION

Accurate diagnosis of *B. pseudomallei* infections is necessary for the prompt administration of effective treatments and for improving the outcomes of melioidosis patients. Due to the lack of highly sensitive, specific, rapid point-of-care tests, and the time-consuming nature of bacterial culture, the diagnosis of melioidosis can be challenging. The 6-deoxyheptan CPS expressed by *B. pseudomallei* has been pursued as a diagnostic target, and several tests use this antigen for the identification of bacterial isolates following bacterial culture and detection of the pathogen in patient samples (5, 14–18, 43). In this study, we report the identification of clinical isolates of *B. cepacia* that express the same CPS antigen as *B. pseudomallei* as determined by reactivity with CPS-specific mAbs in multiple different assays. Total carbohydrate preparations extracted from these strains were positive for CPS using an LFI as well as Western immunoblots. IFAs confirmed the expression of CPS on the surface of the latex-positive *B. cepacia* isolates. Furthermore, whole genome sequencing revealed the presence of homologs of the *B. pseudomallei* CPS biosynthetic genes in the latex-positive *B. cepacia* strains.

Since there is evidence of frequent horizontal gene transfer within and between *Burkholderia* species, the occurrence of serological cross-reactivity between isolates within the genus *Burkholderia* is perhaps not surprising, nor is it unexpected to find that cross-reacting strains from the environment may occasionally be detected in human samples, although this is the first time to our knowledge that this has been reported. This potential cross-reactivity, even when using reagents designed to be specific for *B. pseudomallei* CPS, is an important pitfall of which anyone working on both environmental and clinical samples should be aware. A failure to identify such isolates at the species level could lead to misleading results in environmental surveys, as it did in our previous study, which mistakenly reported the isolation of *B. pseudomallei* from the environment in Luang Namtha Province (44, 46).

In the context of clinical samples, the consequences can be even more significant, with the possibility of inappropriate treatment being given to patients. Fortunately, this phenomenon appears to be restricted to a minority of isolates of *B. cepacia* and *B. thailandensis*, although further surveillance is important to monitor this. Since the initial identification of this phenomenon, the Mahosot Microbiology Laboratory has only isolated one further *B. cepacia* and one *B. thailandensis* from clinical samples that have shown genuine cross-reactivity in the latex agglutination test, along with a number of other isolates of various species that have given non-specific agglutination (i.e., agglutinate with latex beads that are not coated with anti-CPS monoclonal antibody). Nonetheless, it is important that staff working in melioidosis-endemic areas are aware of this phenomenon and do not rely on CPS-based assays such as latex-agglutination, AMD, or IFA alone for the identification of *B. pseudomallei*.

Fortunately, *B. pseudomallei*-specific PCR-based diagnostics are available that are rapid, sensitive, and have high discriminatory power (47–51). The most widely used single target PCR assay that can differentiate between *B. pseudomallei*, *B. thailandensis*, *B. mallei,* and *B. cenocepacia* is based on open reading frame 2 (*orf2*) of the cluster 1 type three secretion system (TTS1) (47, 49, 51, 52). TTS1-*orf2* PCR is highly specific for *B. pseudomallei* and has demonstrated a specificity of 99–100% when used for testing clinical samples (53, 54). Recently identified targets including BPS0745, BPSS1187, and BPSS1498 have also been evaluated in comparison to TTS1-*orf2* in real-time PCR assays, and all were shown to be highly specific for the detection of *B. pseudomallei* (50, 53, 55). Based on this, PCR-based diagnostics should be useful for differentiating *B. pseudomallei* isolates from CPS-expressing *B. cepacia* isolates.

Collectively, the results obtained in this study provide compelling evidence that *B. cepacia* strains expressing the same CPS as *B. pseudomallei* co-exist in the environment alongside *B. pseudomallei* and *B. thailandensis*. Future studies will be necessary to investigate the clinical significance of the CPS-expressing *B. cepacia* isolates and whether or not CPS expression in these strains might enhance their virulence in animal models of infection. In addition, studies aimed at isolating the CPS from these strains for structural determination will be needed to confirm the exact chemical composition of the antigen.

## Data Availability

The genome sequences for the *B. thailandensis* and *B. cepacia* strains described in this study were submitted to GenBank under accession numbers SAMN08724753, SAMN08724754, SAMN08724755, SAMN08724756, and SAMN08724757. Raw sequence data were submitted to the SRA under accession numbers SRS20034251, SRS20041683, SRS20034250, SRS20034253, and SRS20034727.

## References

[1] Compant S, Nowak J, Coenye T, Clément C, Ait Barka E. 2008. Diversity and occurrence of Burkholderia spp. in the natural environment. FEMS Microbiol Rev 32:607–626. doi:10.1111/j.1574-6976.2008.00113.x 18422616

[2] Limmathurotsakul D, Kanoksil M, Wuthiekanun V, Kitphati R, deStavola B, Day NPJ, Peacock SJ, Small PLC. 2013. Activities of daily living associated with acquisition of melioidosis in northeast Thailand: a matched case-control study. PLoS Negl Trop Dis 7:e2072. doi:10.1371/journal.pntd.0002072 23437412 PMC3578767

[3] Wiersinga WJ, Virk HS, Torres AG, Currie BJ, Peacock SJ, Dance DAB, Limmathurotsakul D. 2018. Melioidosis. Nat Rev Dis Primers 4:17107. doi:10.1038/nrdp.2017.107 29388572 PMC6456913

[4] Limmathurotsakul D, Jamsen K, Arayawichanont A, Simpson JA, White LJ, Lee SJ, Wuthiekanun V, Chantratita N, Cheng A, Day NPJ, Verzilli C, Peacock SJ. 2010. Defining the true sensitivity of culture for the diagnosis of melioidosis using bayesian latent class models. PLoS One 5:e12485. doi:10.1371/journal.pone.0012485 20830194 PMC2932979

[5] Duval BD, Elrod MG, Gee JE, Chantratita N, Tandhavanant S, Limmathurotsakul D, Hoffmaster AR. 2014. Evaluation of a latex agglutination assay for the identification of Burkholderia pseudomallei and Burkholderia mallei. Am J Trop Med Hyg 90:1043–1046. doi:10.4269/ajtmh.14-0025 24710616 PMC4047727

[6] Songsri J, Kinoshita Y, Kwanhian W, Wisessombat S, Tangpong J, Rahman-Khan MS, Tuanyok A. 2018. Cross-reactivity of latex agglutination assay complicates the identification of Burkholderia pseudomallei from soil. FEMS Microbiol Lett 365:22. doi:10.1093/femsle/fny256 30346523

[7] Holden MTG, Titball RW, Peacock SJ, Cerdeño-Tárraga AM, Atkins T, Crossman LC, Pitt T, Churcher C, Mungall K, Bentley SD, et al.. 2004. Genomic plasticity of the causative agent of melioidosis, Burkholderia pseudomallei. Proc Natl Acad Sci U S A 101:14240–14245. doi:10.1073/pnas.0403302101 15377794 PMC521101

[8] Reckseidler SL, DeShazer D, Sokol PA, Woods DE. 2001. Detection of bacterial virulence genes by subtractive hybridization: identification of capsular polysaccharide of Burkholderia pseudomallei as a major virulence determinant. Infect Immun 69:34–44. doi:10.1128/IAI.69.1.34-44.2001 11119486 PMC97852

[9] Reckseidler-Zenteno SL, DeVinney R, Woods DE. 2005. The capsular polysaccharide of Burkholderia pseudomallei contributes to survival in serum by reducing complement factor C3b deposition. Infect Immun 73:1106–1115. doi:10.1128/IAI.73.2.1106-1115.2005 15664954 PMC547107

[10] Biryukov SS, Cote CK, Klimko CP, Dankmeyer JL, Rill NO, Shoe JL, Hunter M, Shamsuddin Z, Velez I, Hedrick ZM, Rosario-Acevedo R, Talyansky Y, Schmidt LK, Orne CE, Fetterer DP, Burtnick MN, Brett PJ, Welkos SL, DeShazer D. 2022. Evaluation of two different vaccine platforms for immunization against melioidosis and glanders. Front Microbiol 13:965518. doi:10.3389/fmicb.2022.965518 36060742 PMC9428723

[11] Burtnick MN, Heiss C, Roberts RA, Schweizer HP, Azadi P, Brett PJ. 2012. Development of capsular polysaccharide-based glycoconjugates for immunization against melioidosis and glanders. Front Cell Infect Microbiol 2:108. doi:10.3389/fcimb.2012.00108 22912938 PMC3419357

[12] Burtnick MN, Shaffer TL, Ross BN, Muruato LA, Sbrana E, DeShazer D, Torres AG, Brett PJ. 2018. Development of subunit vaccines that provide high-level protection and sterilizing immunity against acute inhalational melioidosis. Infect Immun 86:e00724-17. doi:10.1128/IAI.00724-17 29109172 PMC5736816

[13] Nelson M, Prior JL, Lever MS, Jones HE, Atkins TP, Titball RW. 2004. Evaluation of lipopolysaccharide and capsular polysaccharide as subunit vaccines against experimental melioidosis. J Med Microbiol 53:1177–1182. doi:10.1099/jmm.0.45766-0 15585494

[14] Nualnoi T, Kirosingh A, Pandit SG, Thorkildson P, Brett PJ, Burtnick MN, AuCoin DP. 2016. In vivo distribution and clearance of purified capsular polysaccharide from Burkholderia pseudomallei in a murine model. PLoS Negl Trop Dis 10:e0005217. doi:10.1371/journal.pntd.0005217 27941991 PMC5179125

[15] Nuti DE, Crump RB, Dwi Handayani F, Chantratita N, Peacock SJ, Bowen R, Felgner PL, Davies DH, Wu T, Lyons CR, Brett PJ, Burtnick MN, Kozel TR, AuCoin DP. 2011. Identification of circulating bacterial antigens by in vivo microbial antigen discovery. mBio 2:e00136-11. doi:10.1128/mBio.00136-11 21846829 PMC3163937

[16] DeMers HL, Nualnoi T, Thorkildson P, Hau D, Hannah EE, Green HR, Pandit SG, Gates-Hollingsworth MA, Boutthasavong L, Luangraj M, Woods KL, Dance D, AuCoin DP. 2022. Detection and quantification of the capsular polysaccharide of Burkholderia pseudomallei in serum and urine samples from melioidosis patients. Microbiol Spectr 10:e0076522. doi:10.1128/spectrum.00765-22 35924843 PMC9430648

[17] Houghton RL, Reed DE, Hubbard MA, Dillon MJ, Chen H, Currie BJ, Mayo M, Sarovich DS, Theobald V, Limmathurotsakul D, Wongsuvan G, Chantratita N, Peacock SJ, Hoffmaster AR, Duval B, Brett PJ, Burtnick MN, Aucoin DP. 2014. Development of a prototype lateral flow immunoassay (LFI) for the rapid diagnosis of melioidosis. PLoS Negl Trop Dis 8:e2727. doi:10.1371/journal.pntd.0002727 24651568 PMC3961207

[18] Woods KL, Boutthasavong L, NicFhogartaigh C, Lee SJ, Davong V, AuCoin DP, Dance DAB. 2018. Evaluation of a rapid diagnostic test for detection of Burkholderia pseudomallei in the Lao people’s democratic republic. J Clin Microbiol 56:e02002-17. doi:10.1128/JCM.02002-17 29720430 PMC6018328

[19] Brett PJ, DeShazer D, Woods DE. 1998. Burkholderia thailandensis sp. nov., a Burkholderia pseudomallei-like species. Int J Syst Bacteriol 48 Pt 1:317–320. doi:10.1099/00207713-48-1-317 9542103

[20] Wuthiekanun V, Smith MD, Dance DA, Walsh AL, Pitt TL, White NJ. 1996. Biochemical characteristics of clinical and environmental isolates of Burkholderia pseudomallei. J Med Microbiol 45:408–412. doi:10.1099/00222615-45-6-408 8958243

[21] Bayliss M, Donaldson MI, Nepogodiev SA, Pergolizzi G, Scott AE, Harmer NJ, Field RA, Prior JL. 2017. Structural characterisation of the capsular polysaccharide expressed by Burkholderia thailandensis strain E555:: wbiI (pKnock-Kmr) and assessment of the significance of the 2-O-acetyl group in immune protection. Carbohydr Res 452:17–24. doi:10.1016/j.carres.2017.09.011 29024844 PMC5697523

[22] Hantrakun V, Thaipadungpanit J, Rongkard P, Srilohasin P, Amornchai P, Langla S, Mukaka M, Chantratita N, Wuthiekanun V, Dance DAB, Day NPJ, Peacock SJ, Limmathurotsakul D. 2018. Presence of B. thailandensis and B. thailandensis expressing B. pseudomallei-like capsular polysaccharide in Thailand, and their associations with serological response to B. pseudomallei. PLoS Negl Trop Dis 12:e0006193. doi:10.1371/journal.pntd.0006193 29364892 PMC5809093

[23] Sim BMQ, Chantratita N, Ooi WF, Nandi T, Tewhey R, Wuthiekanun V, Thaipadungpanit J, Tumapa S, Ariyaratne P, Sung W-K, Sem XH, Chua HH, Ramnarayanan K, Lin CH, Liu Y, Feil EJ, Glass MB, Tan G, Peacock SJ, Tan P. 2010. Genomic acquisition of a capsular polysaccharide virulence cluster by non-pathogenic Burkholderia isolates. Genome Biol 11:R89. doi:10.1186/gb-2010-11-8-r89 20799932 PMC2945791

[24] Glass MB, Gee JE, Steigerwalt AG, Cavuoti D, Barton T, Hardy RD, Godoy D, Spratt BG, Clark TA, Wilkins PP. 2006. Pneumonia and septicemia caused by Burkholderia thailandensis in the United States. J Clin Microbiol 44:4601–4604. doi:10.1128/JCM.01585-06 17050819 PMC1698378

[25] Knappik M, Dance DAB, Rattanavong S, Pierret A, Ribolzi O, Davong V, Silisouk J, Vongsouvath M, Newton PN, Dittrich S. 2015. Evaluation of molecular methods to improve the detection of Burkholderia pseudomallei in soil and water samples from Laos. Appl Environ Microbiol 81:3722–3727. doi:10.1128/AEM.04204-14 25819969 PMC4421066

[26] Wiersinga WJ, Birnie E, Weehuizen TAF, Alabi AS, Huson MAM, Huis in ’t Veld RAG, Mabala HK, Adzoda GK, Raczynski-Henk Y, Esen M, Lell B, Kremsner PG, Visser CE, Wuthiekanun V, Peacock SJ, van der Ende A, Limmathurotsakul D, Grobusch MP. 2015. Clinical, environmental, and serologic surveillance studies of melioidosis in Gabon, 2012-2013. Emerg Infect Dis 21:40–47. doi:10.3201/eid2101.140762 25530077 PMC4285261

[27] Gee JE, Elrod MG, Gulvik CA, Haselow DT, Waters C, Liu L, Hoffmaster AR. 2018. Burkholderia thailandensis isolated from infected wound, Arkansas, USA. Emerg Infect Dis 24:2091–2094. doi:10.3201/eid2411.180821 30334705 PMC6199988

[28] Lertpatanasuwan N, Sermsri K, Petkaseam A, Trakulsomboon S, Thamlikitkul V, Suputtamongkol Y. 1999. Arabinose-positive Burkholderia pseudomallei infection in humans: case report. Clin Infect Dis 28:927–928. doi:10.1086/517253 10825075

[29] Zueter AM, Abumarzouq M, Yusof MI, Wan Ismail WF, Harun A. 2017. Osteoarticular and soft-tissue melioidosis in Malaysia: clinical characteristics and molecular typing of the causative agent. J Infect Dev Ctries 11:28–33. doi:10.3855/jidc.7612 28141587

[30] Anuntagool N, Naigowit P, Petkanchanapong V, Aramsri P, Panichakul T, Sirisinha S. 2000. Monoclonal antibody-based rapid identification of Burkholderia pseudomallei in blood culture fluid from patients with community-acquired septicaemia. J Med Microbiol 49:1075–1078. doi:10.1099/0022-1317-49-12-1075 11129718

[31] Wuthiekanun V, Amornchai P, Saiprom N, Chantratita N, Chierakul W, Koh GCKW, Chaowagul W, Day NPJ, Limmathurotsakul D, Peacock SJ. 2011. Survey of antimicrobial resistance in clinical Burkholderia pseudomallei isolates over two decades in northeast Thailand. Antimicrob Agents Chemother 55:5388–5391. doi:10.1128/AAC.05517-11 21876049 PMC3195054

[32] Edwards U, Rogall T, Blöcker H, Emde M, Böttger EC. 1989. Isolation and direct complete nucleotide determination of entire genes. Characterization of a gene coding for 16S ribosomal RNA. Nucleic Acids Res 17:7843–7853. doi:10.1093/nar/17.19.7843 2798131 PMC334891

[33] Mahenthiralingam E, Bischof J, Byrne SK, Radomski C, Davies JE, Av-Gay Y, Vandamme P. 2000. DNA-based diagnostic approaches for identification of Burkholderia cepacia complex, Burkholderia vietnamiensis, Burkholderia multivorans, Burkholderia stabilis, and Burkholderia cepacia genomovars I and III. J Clin Microbiol 38:3165–3173. doi:10.1128/JCM.38.9.3165-3173.2000 10970351 PMC87345

[34] Turton JF, Arif N, Hennessy D, Kaufmann ME, Pitt TL. 2007. Revised approach for identification of isolates within the Burkholderia cepacia complex and description of clinical isolates not assigned to any of the known genomovars. J Clin Microbiol 45:3105–3108. doi:10.1128/JCM.00976-07 17626169 PMC2045300

[35] Suttisunhakul V, Pumpuang A, Ekchariyawat P, Wuthiekanun V, Elrod MG, Turner P, Currie BJ, Phetsouvanh R, Dance DAB, Limmathurotsakul D, Peacock SJ, Chantratita N. 2017. Matrix-assisted laser desorption/Ionization time-of-flight mass spectrometry for the identification of Burkholderia pseudomallei from Asia and Australia and differentiation between Burkholderia species. PLoS One 12:e0175294. doi:10.1371/journal.pone.0175294 28384252 PMC5383291

[36] Brett PJ, Burtnick MN, Heiss C, Azadi P, DeShazer D, Woods DE, Gherardini FC. 2011. Burkholderia thailandensis oacA mutants facilitate the expression of Burkholderia mallei-like O polysaccharides. Infect Immun 79:961–969. doi:10.1128/IAI.01023-10 21115721 PMC3028842

[37] Burtnick MN, Heiss C, Schuler AM, Azadi P, Brett PJ. 2012. Development of novel O-polysaccharide based glycoconjugates for immunization against glanders. Front Cell Inf Microbio 2:148. doi:10.3389/fcimb.2012.00148 PMC3506924 23205347

[38] Fomsgaard A, Freudenberg MA, Galanos C. 1990. Modification of the silver staining technique to detect lipopolysaccharide in polyacrylamide gels. J Clin Microbiol 28:2627–2631. doi:10.1128/jcm.28.12.2627-2631.1990 1704012 PMC268246

[39] Chin C-S, Alexander DH, Marks P, Klammer AA, Drake J, Heiner C, Clum A, Copeland A, Huddleston J, Eichler EE, Turner SW, Korlach J. 2013. Nonhybrid, finished microbial genome assemblies from long-read SMRT sequencing data. Nat Methods 10:563–569. doi:10.1038/nmeth.2474 23644548

[40] Myers EW, Sutton GG, Delcher AL, Dew IM, Fasulo DP, Flanigan MJ, Kravitz SA, Mobarry CM, Reinert KH, Remington KA, et al.. 2000. A whole-genome assembly of drosophila. Science 287:2196–2204. doi:10.1126/science.287.5461.2196 10731133

[41] Johnson SL, Bishop-Lilly KA, Ladner JT, Daligault HE, Davenport KW, Jaissle J, Frey KG, Koroleva GI, Bruce DC, Coyne SR, Broomall SM, Li P-E, Teshima H, Gibbons HS, Palacios GF, Rosenzweig CN, Redden CL, Xu Y, Minogue TD, Chain PS. 2015. Complete genome sequences for 59 Burkholderia isolates, both pathogenic and near neighbor. Genome Announc 3:e00159-15. doi:10.1128/genomeA.00159-15 25931592 PMC4417688

[42] Galens K, Orvis J, Daugherty S, Creasy HH, Angiuoli S, White O, Wortman J, Mahurkar A, Giglio MG. 2011. The IGS standard operating procedure for automated prokaryotic annotation. Stand Genomic Sci 4:244–251. doi:10.4056/sigs.1223234 21677861 PMC3111993

[43] Tandhavanant S, Wongsuvan G, Wuthiekanun V, Teerawattanasook N, Day NPJ, Limmathurotsakul D, Peacock SJ, Chantratita N. 2013. Monoclonal antibody-based immunofluorescence microscopy for the rapid identification of Burkholderia pseudomallei in clinical specimens. Am J Trop Med Hyg 89:165–168. doi:10.4269/ajtmh.13-0066 23716405 PMC3748476

[44] Rattanavong S, Wuthiekanun V, Langla S, Amornchai P, Sirisouk J, Phetsouvanh R, Moore CE, Peacock SJ, Buisson Y, Newton PN. 2011. Randomized soil survey of the distribution of Burkholderia pseudomallei in rice fields in Laos. Appl Environ Microbiol 77:532–536. doi:10.1128/AEM.01822-10 21075883 PMC3020526

[45] Ho C-C, Lau CCY, Martelli P, Chan S-Y, Tse CWS, Wu AKL, Yuen K-Y, Lau SKP, Woo PCY. 2011. Novel pan-genomic analysis approach in target selection for multiplex PCR identification and detection of Burkholderia pseudomallei, Burkholderia thailandensis, and Burkholderia cepacia complex species: a proof-of-concept study. J Clin Microbiol 49:814–821. doi:10.1128/JCM.01702-10 21177905 PMC3067743

[46] Dance DAB, Luangraj M, Rattanavong S, Sithivong N, Vongnalaysane O, Vongsouvath M, Newton PN. 2018. Melioidosis in the Lao People’s Democratic Republic. Trop Med Infect Dis 3:21. doi:10.3390/tropicalmed3010021 30274419 PMC6136615

[47] Gassiep I, Burnard D, Bauer MJ, Norton RE, Harris PN. 2021. Diagnosis of melioidosis: the role of molecular techniques. Future Microbiol 16:271–288. doi:10.2217/fmb-2020-0202 33595347

[48] Kaestli M, Richardson LJ, Colman RE, Tuanyok A, Price EP, Bowers JR, Mayo M, Kelley E, Seymour ML, Sarovich DS, Pearson T, Engelthaler DM, Wagner DM, Keim PS, Schupp JM, Currie BJ. 2012. Comparison of TaqMan PCR assays for detection of the melioidosis agent Burkholderia pseudomallei in clinical specimens. J Clin Microbiol 50:2059–2062. doi:10.1128/JCM.06737-11 22442327 PMC3372170

[49] Novak RT, Glass MB, Gee JE, Gal D, Mayo MJ, Currie BJ, Wilkins PP. 2006. Development and evaluation of a real-time PCR assay targeting the type III secretion system of Burkholderia pseudomallei. J Clin Microbiol 44:85–90. doi:10.1128/JCM.44.1.85-90.2006 16390953 PMC1351940

[50] Supaprom C, Wang D, Leelayuwat C, Thaewpia W, Susaengrat W, Koh V, Ooi EE, Lertmemongkolchai G, Liu Y. 2007. Development of real-time PCR assays and evaluation of their potential use for rapid detection of Burkholderia pseudomallei in clinical blood specimens. J Clin Microbiol 45:2894–2901. doi:10.1128/JCM.00291-07 17634296 PMC2045258

[51] Thibault FM, Valade E, Vidal DR. 2004. Identification and discrimination of Burkholderia pseudomallei, B. mallei, and B.thailandensis by real-time PCR targeting type III secretion system genes. J Clin Microbiol 42:5871–5874. doi:10.1128/JCM.42.12.5871-5874.2004 15583328 PMC535269

[52] Aung NM, Su KK, Chantratita N, Tribuddharat C. 2023. Workflow for identification of Burkholderia pseudomallei clinical isolates in Myanmar. Jpn J Infect Dis 76:106–112. doi:10.7883/yoken.JJID.2022.508 36450576

[53] Noparatvarakorn C, Jakkul W, Seng R, Tandhavanant S, Ottiwet O, Janon R, Saikong W, Chantratita N. 2023. Optimization and prospective evaluation of sensitive real-time PCR assays with an internal control for the diagnosis of melioidosis in Thailand. Microbiol Spectr 11:e0103923. doi:10.1128/spectrum.01039-23 37819125 PMC10715024

[54] Noparatvarakorn C, Sengyee S, Yarasai A, Phunpang R, Dulsuk A, Ottiwet O, Janon R, Morakot C, Burtnick MN, Brett PJ, West TE, Chantratita N. 2023. Prospective analysis of antibody diagnostic tests and TTS1 real-time PCR for diagnosis of melioidosis in areas where it is endemic. J Clin Microbiol 61:e0160522. doi:10.1128/jcm.01605-22 36877019 PMC10035309

[55] Göhler A, Trung TT, Hopf V, Kohler C, Hartleib J, Wuthiekanun V, Peacock SJ, Limmathurotsakul D, Tuanyok A, Steinmetz I. 2017. Multitarget quantitative PCR improves detection and predicts cultivability of the pathogen Burkholderia pseudomallei. Appl Environ Microbiol 83:e03212-16. doi:10.1128/AEM.03212-16 28188208 PMC5377509

